# Ligation of the internal jugular vein increased regional cerebral oxygen saturation but decreased the bispectral index in a 72-year-old man: a case report

**DOI:** 10.1186/s40981-015-0027-0

**Published:** 2016-01-15

**Authors:** Maiko Hasegawa-Moriyama, Kohei Godai, Tomotsugu Yamada, Akira Matsunaga, Yuichi Kanmura

**Affiliations:** Department of Anesthesiology and Critical Care Medicine, Graduate School of Medical and Dental Sciences, Kagoshima University, 8-35-1 Sakuragaoka, Kagoshima, 890-8520 Japan

**Keywords:** Regional saturation of oxygen, Bispectral index, Internal jugular vein

## Abstract

Regional cerebral oxygen saturation (rSO_2_) and the bispectral index (BIS) are used to detect cerebral perfusion abnormalities. However, whether rSO_2_ and BIS values change during ligation of the internal jugular vein (IJV) is unknown. We report a case in which BIS values were decreased, despite increased rSO_2_ during ligation of the IJV. A 72-year-old man was diagnosed with metastasis of renal cancer to the thyroid associated with tumor embolism in the right IJV. Thyroidectomy with total laryngectomy was performed. After right IJV ligation, right rSO_2_ was increased from 73 to 78 %, while the right BIS value was decreased from 40 to 27. Contralateral rSO_2_ and BIS values were unchanged. Right rSO_2_ and BIS values returned to pre-ligation values in 10 min. Ligation of the IJV might increase cerebral blood flow and ipsilateral rSO_2_. Physicians should use BIS values with caution during IJV ligation because a sudden decrease in the BIS value is not always associated with cerebral hypoperfusion.

## Background

Monitoring of cerebral perfusion during anesthesia is challenging. Regional cerebral oxygen saturation (rSO_2_) and the bispectral index (BIS) can be used to detect cerebral perfusion abnormalities [1–3]. However, whether rSO_2_ and BIS values change during ligation of the internal jugular vein (IJV) is unknown. We report here a case in which BIS values were decreased, despite increased rSO_2_ during ligation of the IJV. Findings in our case suggest that a sudden decrease in BIS value is not always associated with cerebral hypoperfusion.

## Case presentation

**Fig. 1:**
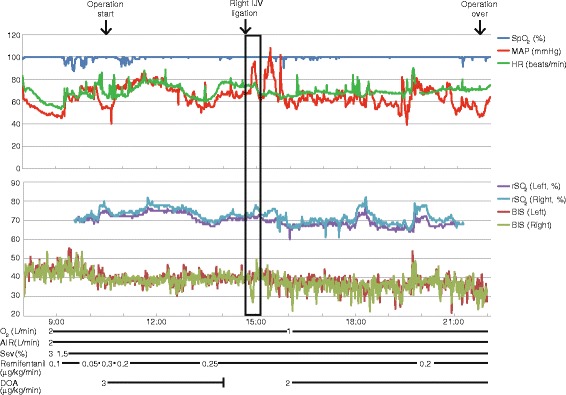
Anesthesia record. Hemodynamic parameters, regional cerebral oxygen saturation (rSO_2_), and the bispectral index (BIS) during general anesthesia are shown. SpO_2_, pulse oximetric oxygen saturation; MAP, mean arterial pressure; HR, heart rate; Sev, sevoflurane; DOA, dopamine

**Fig. 2:**
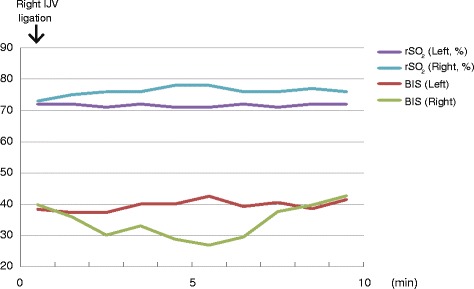
rSO_2_ and BIS values after ligation of the IJV. Changes in regional cerebral oxygen saturation (rSO_2_) and bispectral index (BIS) 10 min after the internal jugular vein (IJV) ligation are shown

A 72-year-old man (160 cm, 48 kg) was diagnosed with metastasis of renal cancer to the thyroid associated with tumor embolism in the right IJV. Computed tomography showed that the tumor had invaded his trachea. The left IJV and vertebral vein plexus were intact. Thyroidectomy with total laryngectomy was scheduled. Five days before surgery, the patient developed breathing difficulties and his trachea was intubated. He was sedated in the intensive care unit until surgery. Anesthesia was induced and maintained with sevoflurane and remifentanil. Dopamine was used to maintain cardiac output. We monitored rSO_2_ with the INVOS OXIMETER (SOMANETICS, Troy, MI, USA) and BIS values with the Bis BILATERAL sensor (Covidien, Minneapolis, MN, USA). After right IJV ligation, right rSO_2_ was increased from 73 to 78 %, while the right BIS value was decreased from 40 to 27 (Figs. 1, 2). Right rSO_2_ and right BIS values returned to pre-ligation values in 10 min. Signal quality index were decreased several minutes during the period, however, there was no significant difference between left and right values (Table 1). Suppression ratio, spectral edge frequency 95, and electromyography remained stable. Although mean arterial pressure increased from 77 to 96 mmHg after ligation of the IJV, contralateral rSO_2_ and BIS values were unchanged. The patient’s respiratory parameters and the depth of general anesthesia remained stable without increased blood loss during this period. The surgical procedure was uneventfully completed and no neurological deficit was observed postoperatively.

**Table 1:** Additional data of BIS values after ligation of the IJV. Changes in suppression ratio (SR), spectral edge frequency 95 (SEF95), electromyography (EMG) and signal quality index (SQI) 10 min after the internal jugular vein (IJV) ligation are shown

Minutes after IJV ligation	SR (Left, %)	SEF95 (Left, Hz)	EMG (Left, dB)	SQI (Left, %)	SR (Right, %)	SEF95 (Right, Hz)	EMG (Right dB)	SQI (Right, %)
1	0	12.8	24.7	94.9	0	12.3	25.4	94.9
2	0	10.4	25.1	87.8	0	10.2	26.4	82.1
3	0	10.4	24.7	62.2	0	10.5	24.9	59.6
4	0	8.6	24.8	55.1	0	8.2	24.8	55.1
5	0	8.2	24.8	94.2	0	7.5	24.6	94.9
6	0	9.2	24.7	83.3	0	8.5	24.9	82.1
7	0	8.2	29.4	84	0	6.9	33.9	78.2
8	0	8.4	24.9	80.8	0	7.8	25.4	79.5
9	0	9.9	24.8	69.2	0	10	24.8	66.7
10	0	10.6	24.4	91.7	0	10.7	24.5	82.7

Two important phenomena were observed in this case. First, the sudden decrease in unilateral BIS value did not appear to be associated with cerebral hypoperfusion. Second, ligation of the IJV increased ipsilateral rSO_2_ and decreased ipsilateral BIS values without any effects in contralateral rSO_2_ and BIS values.

In this case, the sudden decrease in BIS value did not indicate cerebral hypoperfusion. Several case reports have indicated that BIS monitoring may be a marker of cerebral hypoperfusion. Morimoto et al. described a case in which the BIS value was 0, possibly because of cerebral hypoperfusion [2]. Kodaka et al. reported that bilateral BIS monitoring can detect cerebral hypoperfusion during carotid endarterectomy [3]. However, another study showed that BIS values did not correlate with cerebral ischemia in 52 patients who underwent carotid endarterectomy [4]. Because the BIS is not a monitor for cerebral blood flow, a low BIS value does not always coincide with cerebral hypoperfusion. Bonhomme et al. showed that BIS values decreased in only 25 % of 36 patients during carotid artery cross clamping [5]. Paradoxical increases in BIS values were observed in 47 % of patients. BIS monitoring should be used with caution during situations in which cerebral perfusion rapidly changes.

In our case, after ligation of the IJV, ipsilateral rSO_2_ was temporarily increased, possibly because of the increase in cerebral blood flow, whereas the ipsilateral BIS value was decreased at that time. Several parameters affect rSO_2_, such as mean arterial pressure, arterial oxygen saturation, partial pressure of carbon dioxide, hemoglobin concentration, and cerebral blood flow [6]. In our case, although mean arterial pressure was significantly increased, the other parameters remained stable. Increased rSO_2_ appeared to be due to increased hemispheric cerebral perfusion because contralateral rSO_2_ did not change. Chai et al. reported that ligation of the right IJV increases cerebral blood flow in anesthetized swine [7]. They also showed that IJV ligation does not increase intracranial pressure or ipsilateral IJV pressure. Ensari et al. described a patient with bilateral internal, external, jugular vein ligations [8]. They showed that the venous drainage route of the brain had been changed from the jugular veins to the vertebral venous plexus after jugular vein ligation. We speculate that in our case, ligation of the right IJV led to increased cerebral blood flow, and blood was drained via the left IJV or vertebral venous plexus without any increase in intracranial pressure.

After right IJV ligation, the very first change in the brain might be the venous congestion. The venous congestion, however, may be resolved within a few minutes since IJV ligation in swine did not lead to increased jugular venous pressure in five minutes [7]. We speculate that ligation of the IJV created an acute decrease in regional cerebral oxygen delivery by changing cerebral blood flow patterns. The acute decrease in regional cerebral oxygen delivery might increase regional cerebral blood flow, which resulted in an increased arterial/venous blood ratio in the right hemisphere. However, changed cerebral blood flow also might change anesthetic concentration only in the right hemisphere. We suppose that the changed anesthetic concentration led to the decreased BIS values. There seems to be fluctuation in BIS values even 20 min after the IJV ligation. We also consider the changed anesthetic concentration was the reason of the fluctuation.

## Conclusions

We here report a case in which BIS values were decreased, despite increased rSO_2_ during ligation of the IJV. Ligation of the IJV may increase cerebral blood flow and ipsilateral rSO_2_. Physicians should use BIS values with caution during IJV ligation because a sudden decrease in the BIS value is not always associated with cerebral hypoperfusion.

## Consent

Written informed consent was obtained from the patient for the publication of this case report and any accompanying images. A copy of the written consent is available for review by the Editor-in-Chief of this journal.

## Abbreviations

BIS: bispectral index
IJV: internal jugular vein
rSO_2_: regional saturation of oxygen
